# Virulence-related factors and antimicrobial resistance in *Proteus mirabilis* isolated from domestic and stray dogs

**DOI:** 10.3389/fmicb.2023.1141418

**Published:** 2023-05-10

**Authors:** Lijuan Liu, Zhiyou Dong, Shengquan Ai, Shanyu Chen, Mengyao Dong, Qianlan Li, Ziyao Zhou, Haifeng Liu, Zhijun Zhong, Xiaoping Ma, Yanchun Hu, Zhihua Ren, Hualin Fu, Gang Shu, Xianmeng Qiu, Guangneng Peng

**Affiliations:** ^1^Key Laboratory of Animal Disease and Human Health of Sichuan, College of Veterinary Medicine, Sichuan Agricultural University, Chengdu, China; ^2^New Ruipeng Pet Healthcare Group, Chengdu, China; ^3^State Key Laboratory of Agricultural Microbiology, National Engineering Research Center of Microbial Pesticides, College of Life Science and Technology, Huazhong Agricultural University, Wuhan, China

**Keywords:** *Proteus mirabilis*, virulence-related factors, antimicrobial resistance, antibiotic resistance genes, virulence-associated genes, domestic and stray dogs

## Abstract

**Introduction:**

*Proteus mirabilis* is a multi-host pathogen that causes diseases of varying severity in a wide range of mammals, including humans. *Proteus mirabilis* is resistant to multiple antibiotics and has acquired the ability to produce expanded spectrum of β-lactamases, leading to serious public health problems. However, the available information on *P. mirabilis* isolated from feces of dogs, is still poorly understood, as is the correlation between its virulence-associated genes (VAGs) and antibiotic resistance genes (ARGs).

**Method:**

In this study, we isolated 75 strains of *P. mirabilis* from 241 samples, and investigated the swarming motility, biofilm formation, antimicrobial resistance (AMR), distribution of VAGs and ARGs, as well as the presence of class 1, 2, and 3 integrons in these isolates.

**Results:**

Our findings suggest a high prevalence of intensive swarming motility and strong biofilm formation ability among *P. mirabilis* isolates. Isolates were primarily resistant to cefazolin (70.67%) and imipenem (70.67%). These isolates were found to carry *ureC*, *FliL*, *ireA*, *zapA*, *ptA*, *hpmA*, *hpmB*, *pmfA*, *rsbA*, *mrpA*, and *ucaA* with varying prevalence levels of 100.00, 100.00, 100.00, 98.67, 98.67, 90.67, 90.67, 90.67, 90.67, 89.33, and 70.67%, respectively. Additionally, the isolates were found to carry *aac(6′)-Ib*, *qnrD*, *floR*, *bla*_CTX-M_, *bla*_CTX-M-2_, *bla*_OXA-1_, *bla*_TEM_, *tetA*, *tetB* and *tetM* with varying prevalence levels of 38.67, 32.00, 25.33, 17.33, 16.00, 10.67, 5.33, 2.67, 1.33, and 1.33%, respectively. Among 40 MDR strains, 14 (35.00%) were found to carry class 1 integrons, 12 (30.00%) strains carried class 2 integrons, while no class 3 integrons was detected. There was a significant positive correlation between the class 1 integrons and three ARGs: *bla*_TEM_, *bla*_CTX-M_, and *bla*_CTX-M-2_. This study revealed that *P. mirabilis* strains isolated from domestic dogs exhibited a higher prevalence of MDR, and carried fewer VAGs but more ARGs compared to those isolated from stay dogs. Furthermore, a negative correlation was observed between VAGs and ARGs.

**Discussion:**

Given the increasing antimicrobial resistance of *P. mirabilis*, veterinarians should adopt a prudent approach towards antibiotics administration in dogs to mitigate the emergence and dissemination of MDR strains that pose a potential threat to public health.

## 1. Introduction

*Proteus mirabilis*, a member of the *Enterobacteriaceae* family, as an opportunistic pathogen can cause skin infection, respiratory tract infection, urinary tract infection (UTI), and gastrointestinal tract infection, and it has been suggested that it may be related to Crohn’s disease (Zhang J. W. et al., 2021). As a zoonotic bacteria, *P. mirabilis* can infect a variety of animals, such as chicken (Wong et al., 2013), ducks (Algammal et al., 2021), turtles (Pathirana et al., 2018), cattle (Sun et al., 2020), companion animals (Marques et al., 2019).

The virulence factors of *P. mirabilis* include flagella, pili, urease, hemolysin, metalloproteinase, which can help *P. mirabilis* to colonize, destroy tissues, and escape immunity (Coker et al., 2000). *Proteus mirabilis* is a model organism for urease^+^ bacteria (Norsworthy and Pearson, 2017). The main functional subunit of urease, the alpha subunit, is encoded by *ureC*, which breaks down urea into one carbonic acid and two ammonia molecules (Armbruster et al., 2018). Ammonia produced by urease can increase the pH of urine above 7.2 and precipitates calcium and magnesium compounds into crystals of magnesium ammonium phosphate (struvite) and calcium phosphate (apatite) (Broomfield et al., 2009). Struvite and apatite crystals are deposited in biofilms to form crystalline biofilms (Armbruster et al., 2018). The presence of crystalline biofilms makes antibiotic treatment more difficult (Maszewska et al., 2021). *Proteus mirabilis* has peritrichous flagella, which can carry out the typical swarming motility, called the “bull-eye” (Aygül et al., 2019). The swarming motility of *P. mirabilis* is a process in which vegetative cells periodically differentiate into swarming cells on solid surfaces.

The swarming motility is important because it is coupled to the expression of virulence-associated genes (VAGs) and the ability to invade cells (Rather, 2005). It has been shown that there is a specific correlation between virulence factors and antimicrobial resistance (AMR) in *P. mirabilis* (Filipiak et al., 2020). The AMR of clinical *P. mirabilis* isolates is gradually increasing. Studies have shown that in addition to intrinsic resistance to tetracycline and polymyxin, *P. mirabilis* has acquired resistance to the β-lactams (Shelenkov et al., 2020). *Proteus mirabilis* is usually susceptible to fluoroquinolones (Girlich et al., 2020). Multidrug-resistant (MDR) was defined as acquired non-susceptibility to at least one agent in three or more antimicrobial categories (Magiorakos et al., 2012). MDR isolates of *P. mirabilis* have been identified in both human and veterinary medicine, emphasizing on the need for continuous surveillance (Decôme et al., 2020). MDR may be mediated by mutations in a resistance gene, or resistance genes may be acquired through horizontal transfer. These resistant genes are widely present on plasmids and integrons, leading to the problems of rapid transmission and treatment failure (Leverstein-van Hall et al., 2003). Recent studies have also shown that the prevalence of β-lactams resistance genes in *P. mirabilis* is gradually increasing (Wong et al., 2013; Algammal et al., 2021). The antibiotic resistance gene (ARG) is one of the many mechanisms by which bacteria can develop AMR (Hughes and Andersson, 2017). However, the presence of a specific ARG does not necessarily lead to the corresponding AMR, as other genetic and environmental factors can influence the expression and function of the gene (Hughes and Andersson, 2017). Previous studies in *Escherichia coli* have revealed both positive and negative associations between VAGs and ARGs, with predominance of positive correlations (Zhao et al., 2021; Zhang S. et al., 2021). But little is known about the AMR, VAGs, and ARGs of *P. mirabilis* isolated from feces of dogs and the correlations among them.

Previous studies have shown that UTI in companion animals and humans may be caused by closely related *P. mirabilis* strains and that such strains from both origins share common ARGs and VAGs (Marques et al., 2019), which suggest that companion animals may serve as potential reservoirs of antibiotic-resistant bacteria in humans. *Proteus mirabilis* also was considered to be the host of storing ARGs and VAGs (Hu et al., 2020). With 50.85 million dogs living in Chinese cities as of 2018, dogs are the most popular companion animals in China (Yin et al., 2020). There have been some studies of *P. mirabilis* isolated from the urinary tract of dogs (Gaastra et al., 1996; Harada et al., 2014). Therefore, there is great significance in monitoring AMR, ARGs and VAGs carried by *P. mirabilis* isolates from dog feces for public health security. Additionally, prior studies overlooked *P. mirabilis* isolates carried by stray dogs. Therefore, this study aimed to determine the prevalence of *P. mirabilis* in the feces of domestic dogs and stray dogs. Furthermore, the swarming motility, biofilm formation, AMR, VAGs, and ARGs of *P. mirabilis* isolates were detected and their correlations were analyzed.

## 2. Materials and methods

### 2.1. Sample collection

From April 2021 to April 2022, we collected a total of 241 dog fecal swabs, comprising of 147 fecal swabs from domestic dogs obtained from 8 pet hospitals situated in Chengdu city, Sichuan province in southwestern China, as well as 94 fecal swabs from stray dogs sourced from the Center of Protect Beastie located in Sichuan Province. All stray dog fecal swabs were collected by laboratory staff and domestic dog fecal swabs were collected by veterinarians. To minimize the risk of contamination between samples, disinfection of hands and changing of disposable gloves were mandatory for staff involved in sample collection. The fecal swabs of the dogs were collected with sterile cotton swabs into sterile Eppendorf (EP) tubes and placed in a foam box with an ice pack, then sent to the laboratory by express as soon as possible for bacteriological examination.

### 2.2. *Proteus mirabilis* screening

The obtained samples were enriched in 1 mL of Luria-Bertani (LB) broth at 37°C, 120 r/min for 24 h. The bacteria suspension was streaked onto LB solid medium using an inoculation loop, and observed for colony morphology on LB agar medium after 24 h of static cultivation at 37°C. Suspected *P. mirabilis* colonies were selected and streaked on *Salmonella-Shigella* (SS) agar medium for further purification. The colony color on SS agar medium was observed, and then the single colony with a black center was selected for Gram staining and observed under microscope.

### 2.3. Identification of isolates

Firstly, biochemical tests such as gelatin liquefaction test, fermentation test of maltose, glucose, sucrose, lactose and mannose, VP test, nitrate dynamic test, MR Test, indole test, iron triosaccharide test, hydrogen sulfide test and citrate utilization were carried out on all isolates using biochemical tubes and interpreted according to Berger’s manual. Then, the 16S rRNA gene was amplified by polymerase chain reaction (PCR) and combined with biochemical result to identify *P. mirabilis* isolates (Gao et al., 2018). The PCR products were then sent to Beijing Tsingke Biotechnology Co., LTD for sequencing. BLAST (Basic Local Alignment Search Tool) was used to detect sequence homology between the sequencing results and GenBank accessions.

### 2.4. Hemolysin and urease production

The hemolysis properties of *P. mirabilis* isolates were determined by observing clear zones around bacterial colonies on blood agar supplemented with 5% defibrinated sheep blood after 24 or 48 h of static cultivation in 37°C. The bacterial suspension of *P. mirabilis* isolates was streaked on SS agar medium and incubated at 37°C for 24 h. A single colony was selected and streaked into the urease biochemical tubes and incubated at 37°C for 8–10 h to observe the color change. A change in color from orange to red observed in the biochemical tubes indicates the production of ureses by isolates.

### 2.5. Swarming motility testing

The swarming motility rates of *P. mirabilis* isolates were assessed by measuring the coverage scale of LB solid medium after incubating under the same conditions. LB solid medium with an agar concentration of 1.5% was prepared. After the medium was solidified, the plates were dried in an oven at 42°C for 60 min before use. The optical density of the bacterial suspension propagated to the logarithmic growth stage was measured at 600 nm and diluted to an optical density (OD) 600 of 0.4. 5 μL of the diluted bacterial suspension was inoculated into the center of LB solid plates. The plates were inverted and incubated at 37°C after the bacterial suspension was absorbed into the agar matrix (~5 min). Swarming motility of *P. mirabilis* isolates was observed and the coverage scale was measured after 9 h. The ability of swarming motility is divided into three categories: category 1, weak swarming (coverage ≤5%); category 2 medium swarming (5% < coverage ≤25%); category 3, dense swarming (coverage >25%; Filipiak et al., 2020). The assay was repeated three times for each strain.

### 2.6. Estimation of the biofilm formation

Biofilm formation was determined by using the crystal violet staining method in 96-well cell plates. After the OD_600_ of the bacterial solution culture to the logarithmic growth stage was detected and adjusted to 0.1, 200 μL diluted bacterial suspension was added to a 96-well cell culture plate and incubated for 24 h at 37°C. The experiment was repeated with 3 wells for each strain. Wells containing only LB broth medium were used as negative controls. After 24 h, the biofilm formed in 96-well cell culture plates was stained by crystal violet staining according to the literature (Sun et al., 2020). After removing the medium and washing the cells with phosphate-buffered saline (PBS), the bacteria were stained with 200 μL crystal violet for 5 min. The crystal violet dye was solubilized by the addition of 200 μL of organic solvent (anhydrous ethanol: acetone = 70:30, v/v), and was quantified by measuring absorbance at 590 nm with a microplate reader. When OD value exceeds 1, dilute with 33% glacial acetic acid, and multiply the obtained value by dilution ratio.

The mean OD of the negative control plus three times its standard deviation (SD) was defined as the cut-off value (ODc). Based on the ODc, the biofilm-forming ability of isolates can be divided into the following four types: OD_590_ ≤ ODc is a non-biofilm-forming strain (−), ODc < OD_590_ ≤ 2ODc is a weak biofilm-forming strain (+), and 2ODc < OD_590_ ≤ 4ODc is medium biofilm-forming strains (++), OD_590_ > 4ODc is strong biofilm-forming strains (+++) (Khoramian et al., 2015).

### 2.7. Antimicrobial susceptibility testing

Kirby-Bauer disc diffusion method was used to evaluate the AR of 75 *P. mirabilis* isolates to 15 antibiotics in 7 categories. These antibiotics include ampicillin (AMP) (10 μg), amoxicillin/clavulanic acid (AUG) (20/10 μg), ampicillin/sulbactam (SAM) (10/10 μg), cefazolin (CZO) (30 μg), cefepime (FEP) (30 μg), cefotaxime (CTX) (30 μg), imipenem (IPM) (10 μg), meropenem (MEM) (10 μg), aztreonam (ATM) (30 μg), gentamicin (GEN) (10 μg), tetracycline (TET) (30 μg), ciprofloxacin (CIP) (5 μg), sulfamethoxazole/trimethoprim (SXT) (25 μg), fosfomycin (FOS) (200 μg) and chloramphenicol (C) (30 μg). In brief, bacterium suspension of 0.5 McFarland was uniformly spread onto Mueller-Hinton (MH) agar plates and incubated at 37°C for 18 h. *Escherichia coli* ATCC 25922 was used as the control microorganism. The inhibitory zone around each disc was measured, and the results were interpreted according to the guidelines provided by the manufacturer and the Clinical and Laboratory Standards Institute (CLSI; Clinical and Laboratory Standards Institute, 2020). Isolates were considered non-susceptible if they were intermediate or resistant to a certain antibiotic (Decôme et al., 2020). MDR was defined as acquired non-susceptibility to at least one agent in three or more antimicrobial categories (Magiorakos et al., 2012). Non-MDR was defined as resistance to none or up to two antimicrobial categories (Qi et al., 2016).

### 2.8. Detection of VAGs, ARGs, and integrons

The distribution of VAGs and ARGs in 75 *P. mirabilis* isolates was investigated through screening of 11 VAGs, 18 ARGs, and class 1, 2, and 3 integrons using conventional PCR. The details of VAGs, ARGs, and integrons (gene names, primer sequences, product lengths and annealing temperatures) are shown in Supplementary Tables 1, 2. The reaction system containing 12.5 μL of 2 × Taq Master Mix, 1 μL each of upstream/downstream primers, 2 μL of bacterial-DNA, and then sterile ddH2O was added up to 25 μL. The cycling conditions of VAGs were as follows: initial degeneration at 94°C for 2 min; followed by 30 cycles of denaturation at 94°C for 2 min, anneal for 1 min and extension at 72°C for 1 min; 72°C, 5 min. The cycling conditions of ARGs and integrons were as follows: initial degeneration at 93°C for 5 min; followed by 30 cycles of denaturation at 93°C for 30s, anneal for 30s and extension at 72°C for 1 min; 72°C 5 min. At the same time, negative controls without DNA were set. PCR products with positive bands after electrophoresis screening were sent to Beijing Tsingke Biotechnology Co., Ltd. for sequencing. BLAST (Basic Local Alignment Search Tool) was used to detect sequence homology between the sequencing results and GenBank accessions.

### 2.9. Statistical analyses

The obtained data were analyzed using the Chi-square test (SPSS software, version 9.4; Significance-level; *p* < 0.05). Comparisons between groups were conducted by Fisher’s Exact test, with an alpha value of 0.05. The statistical analysis took into account standard deviations, and they are based on data obtained from repeated experiments.

## 3. Results

### 3.1. Prevalence of *Proteus mirabilis* in the examined samples

A total of 75 non-duplicate strains of *P. mirabilis* were isolated from 241 samples of dog feces, and the isolation rate was 31.12% (75/241). Among them, 41 strains of *P. mirabilis* isolates were from domestic dog feces collected from pet hospitals, and 34 strains of *P. mirabilis* isolates were from stray dog feces collected from the Center of Protect Beastie (Table 1). The detection rate of *P. mirabilis* in the feces of stray dogs was significantly higher than that of domestic dogs (*p* < 0.01).

**Table 1:** Prevalence of *Proteus mirabilis* in dog fecal samples (*N* = 241).

Sources of samples	Prevalence of *P. mirabilis* (*n*)	*p*-value
Domestic dogs	27.89% (41/147)	0.004^**^
Stray dogs	36.17% (34/94)	
Total	30.71% (75/241)	

** represents a highly significant difference.

### 3.2. Hemolysin and urease production

On defibrinated sheep blood agar plates, all *P. mirabilis* isolates produced color changes, but none exhibited typical β-hemolysis. After 8–10 h of incubation, the urease biochemical tubes inoculated with *P. mirabilis* isolates changed from orange yellow to rose red, indicating that all 75 strains of *P. mirabilis* isolates could produce urease to decompose urea.

### 3.3. Swarming motility testing

In this experiment, all *P. mirabilis* isolates showed the ability of “fog creep” migration. The isolate with the strongest ability of swarming motility was PM55 with a velocity of 5.43 mm/h, while the isolate with the weakest ability of swarming motility was PM13, whose speed was only 0.125 mm/h. Additionally, 7 (6.67%) isolates of *P. mirabilis* exhibited weak swarming motility; 32 (42%) isolates exhibited moderate swarming motility, and 38 (50.67%) isolates exhibited strong swarming motility. There was no significant difference in swarming motility between *P. mirabilis* isolated from domestic and stray dog feces (*p* > 0.05).

### 3.4. Estimation of the biofilm formation

The test of biofilm formation showed that all *P. mirabilis* isolates could form biofilm. Only 7 strains (9.33%) of *P. mirabilis* isolates were medium biofilm producers (0.7 < OD_590_ ≤ 1.4), and 68 strains (90.67%) are intensive biofilm producers (OD_590_ > 1.4; Supplementary Figure 3). There was no significant difference in biofilm formation between *P. mirabilis* isolated from domestic and stray dog feces (*p* > 0.05).

### 3.5. Antimicrobial susceptibility

The AMR of the *P. mirabilis* isolates was assessed and is presented in Table 2. The results indicate that the resistance rate of these *P. mirabilis* isolates to tetracycline (TE) was the highest (75, 100%), followed by cefazolin (KZ) (53, 70.67%) and imipenem (IPM) (53, 70.67%), ampicillin (AMP) (27, 36.00%), fosfomycin (FOS) (26, 34.67%), sulfamethoxazole/trimethoprim (SXT) (23, 30.67%), meropenem (MEM) (22, 29.33%), chloramphenicol (C) (21, 28.00%), cefepime (FEP) (17, 22.67%), ciprofloxacin (CIP) (15, 20.00%), cefotaxime (CTX) (13, 17.33%), amoxicillin/clavulanic acid (AMC) (11, 14.67%), ampicillin/sulbactam (SAM) (11, 14.67%), gentamicin (CN) (9, 12%), aztreonam (ATM) (1, 1.33%). Moreover, the AMR of *P. mirabilis* isolates from the two sources was significantly different to different antibiotics (*p* < 0.05; Supplementary Figure 4).

**Table 2:** Antimicrobial resistance in *Proteus mirabilis* isolates.

Antimicrobial category		Antibiotics agents	Percentage of antibiotic resistant strains (*n*)	Percentage of antibiotic resistant strains from different sources		*p*-value
				Domestic dogs (*n*)	Stray dogs (*n*)	
β-lactams	Penicillins	Ampicillin (AMP)	36.00% (27/75)	58.54% (24/41)	8.82% (3/34)	<0.001^***^
	β-lactam compound	Amoxicillin/clavulanic acid (AMC)	14.67% (11/75)	26.83% (11/41)	0% (0/34)	<0.001^***^
		Ampicillin/sulbactam (SAM)	14.67% (11/75)	26.83% (11/41)	0% (0/34)	<0.001^***^
	Cephalosporins	Cefazolin (KZ)	70.67% (53/75)	87.80% (36/41)	50.00% (17/34)	<0.001^***^
		Cefepime (FEP)	22.67% (17/75)	29.27% (12/41)	14.71% (5/34)	0.134
		Cefotaxime (CTX)	17.33% (13/75)	31.71% (13/41)	0% (0/34)	<0.001^***^
	Carbapenems	Imipenem (IPM)	70.67% (53/75)	58.54% (24/41)	85.29% (29/34)	0.011^*^
		Meropenem (MEM)	29.33% (22/75)	21.95% (9/41)	38.24% (13/34)	0.123
	Monocyclic lactam	Aztreonam (ATM)	1.33% (1/75)	2.44% (1/41)	0% (0/34)	0.547
Aminoglycosides		Gentamicin (CN)	12.00% (9/75)	21.95% (9/41)	0% (0/34)	0.003^**^
Tetracyclines		Tetracycline (TE)	100% (75/75)	100% (41/41)	100% (34/34)	-
Quinolones		Ciprofloxacin (CIP)	20.00% (15/75)	36.59% (15/41)	0% (0/34)	<0.001^***^
Sulfonamides		Sulfamethoxazole/trimethoprim (SXT)	30.67% (23/75)	51.22% (21/41)	5.88% (2/34)	<0.001^***^
Polyphosphates		Fosfomycin (FOS)	34.67% (26/75)	34.15% (14/41)	35.29% (12/34)	0.917
Styrene acrylic alcohols		Chloramphenicol (C)	28.00% (21/75)	51.22% (21/41)	0% (0/34)	<0.001^***^

Specific antimicrobial resistance phenotypes of *P. mirabilis* isolates are shown in Supplementary Table 1. * represents a significant difference, ** represents a highly significant difference, and *** denotes an extremely significant difference.

Out of 75 *P. mirabilis* isolates, 40 strains (53.33%) with MDR were identified, comprising of 30 strains sourced from domestic dogs and 10 from stray dogs (Table 3). *Proteus mirabilis* strains isolated from domestic dogs exhibited a significantly higher prevalence of MDR than those isolated from stray dogs (*p* < 0.001).

**Table 3:** Percentage of MDR *Proteus mirabilis* isolates from two sources.

The sources of samples	MDR (*N* = 40)	non-MDR (*N* = 35)	*p*-value
Domestic dogs	75.00% (30/40)	31.43% (11/35)	<0.001^***^
Stray dogs	25.00% (10/40)	68.57% (24/35)	
Total	53.33% (40/75)	46.67% (35/75)	

*** denotes an extremely significant difference.

### 3.6. Detection of VAGs, ARGs, and integrons

**Figure 1:**
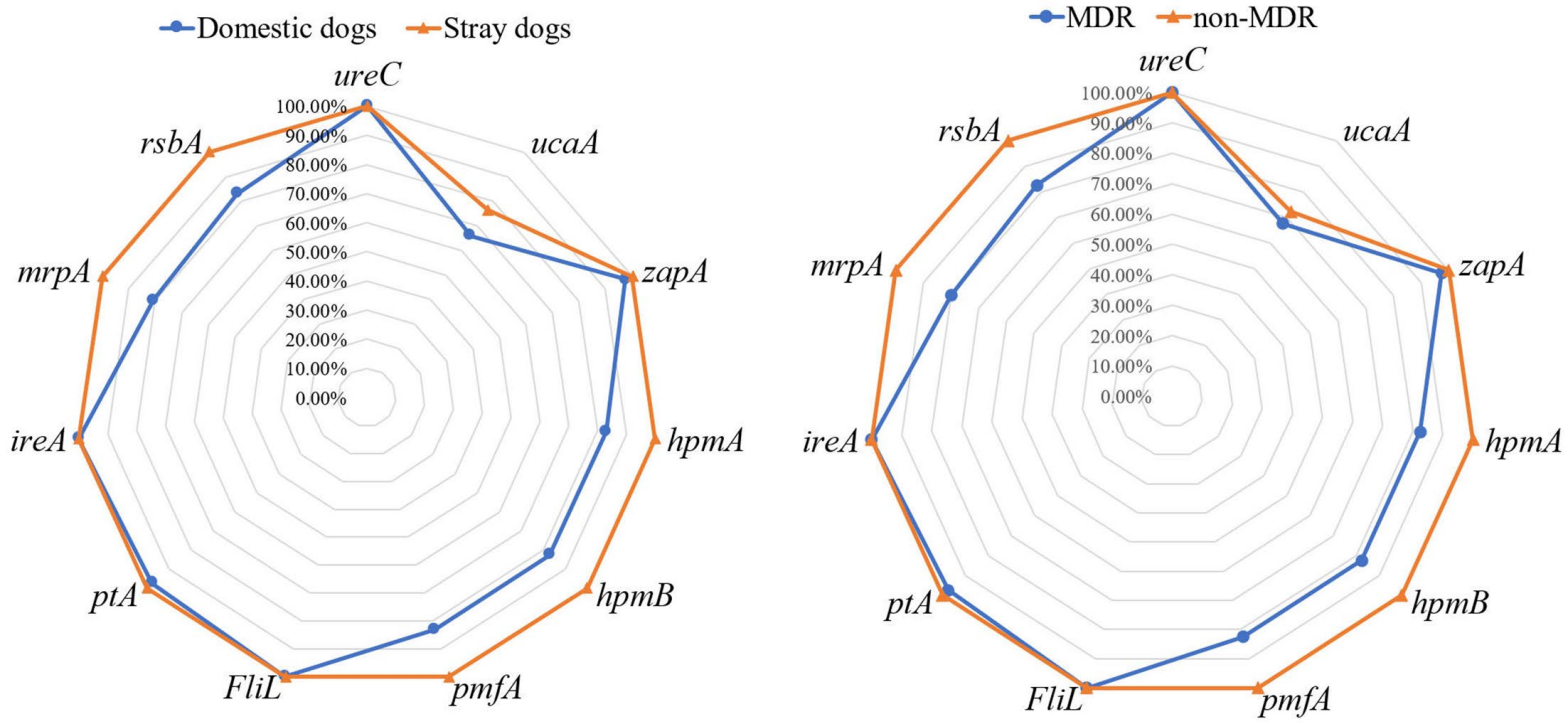
The prevalence of virulence-associated genes in *Proteus mirabilis* isolates. The radar map on the left shows the virulence-associated genes carried by *P. mirabilis* strains from domestic and stray dogs. The radar map on the right shows the virulence-associated genes carried by MDR and non-MDR strains.

This study identified 11 VAGs in 75 *P. mirabilis* isolates. Figure 1 shows that *ureC*, *FliL* and *ireA* were the universally present VAGs with a detection rate of 100%, followed by *zapA* (98.67%), *ptA* (98.67%), *hpmA* (90.67%), *hpmB* (90.67%), *pmfA* (90.67%), *rsbA* (90.67%), *mrpA* (89.33%) and *ucaA* (70.67%). The prevalence rates of VAGs (*hpmA*, *hpmB*, *pmfA*, *mrpA*, and *rsbA*) of *P. mirabilis* strains isolated from stray dogs were significantly higher than those isolated from domestic dogs (*p* < 0.05). Furthermore, the prevalence rates of these VAGs in non-MDR strains were significantly higher than those in MDR strains (*p* < 0.05).

**Figure 2:**
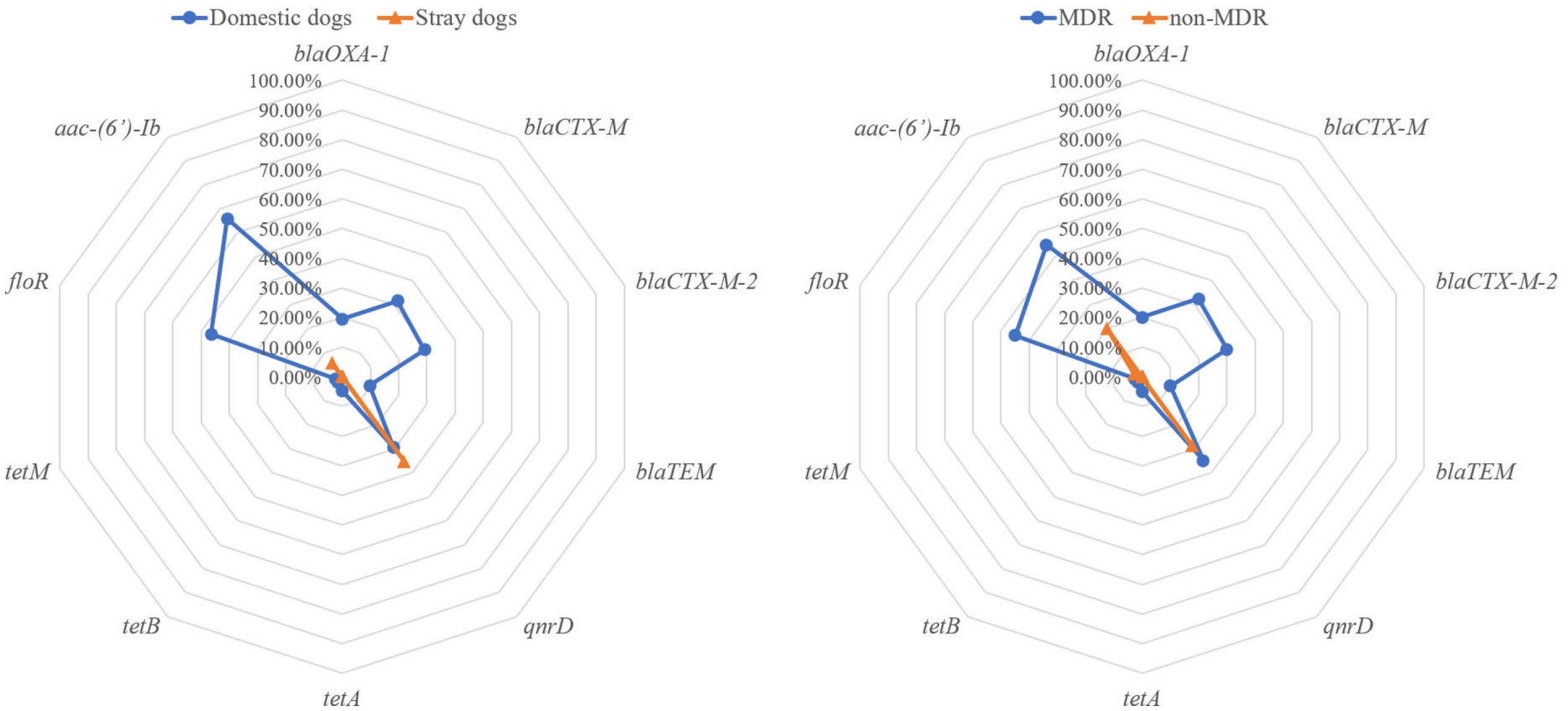
The prevalence of antibiotic resistance genes of the *Proteus mirabilis* isolates. The radar map on the left shows the antibiotic resistance genes carried by *P. mirabilis* strains from domestic and stray dogs. The radar map on the right shows the antibiotic resistance genes carried by MDR and non-MDR strains.

In total, 10 out of 18 ARGs were identified in 75 *P. mirabilis* isolates. Among these, aac-(6′)-Ib was the universally present ARG with a prevalence rate of 38.67%, followed by *qnrD* (32.00%), *floR* (25.33%,), *bla*_CTX-M_ (17.33%), *bla*_CTX-M-2_ (16.00%), *bla*_OXA-1_ (10.67%), *bla*_TEM_ (5.33%), *tetA* (2.67%), *tetB* (1.33%) and *tetM* (1.33%; Figure 2). The detection rates of ARGs (*bla*_OXA-1_, *bla*_CTX-M_, *bla*_CTX-M-2_, *floR* and *aac-(6′)-Ib*) in *P. mirabilis* isolated from domestic dogs were significantly higher when compared to those isolated from stray dogs (*p* < 0.01). Furthermore, the detection rates of these ARGs in MDR strains were significantly higher when compared to non-MDR strains (*p* < 0.01). Out of 40 MDR strains, 14 (35.00%) were found to carry class 1 integrons, 12 (30.00%) strains carried class 2 integrons, while no class 3 integrons was detected. The abundance of the class 2 integrons was found to be significantly higher in *P. mirabilis* isolates from domestic dogs compared to that from stray dogs (*p* < 0.05), while no significant difference in class 1 integrons was observed between stray and domestic dogs.

### 3.7. Correlations analysis

**Figure 3:**
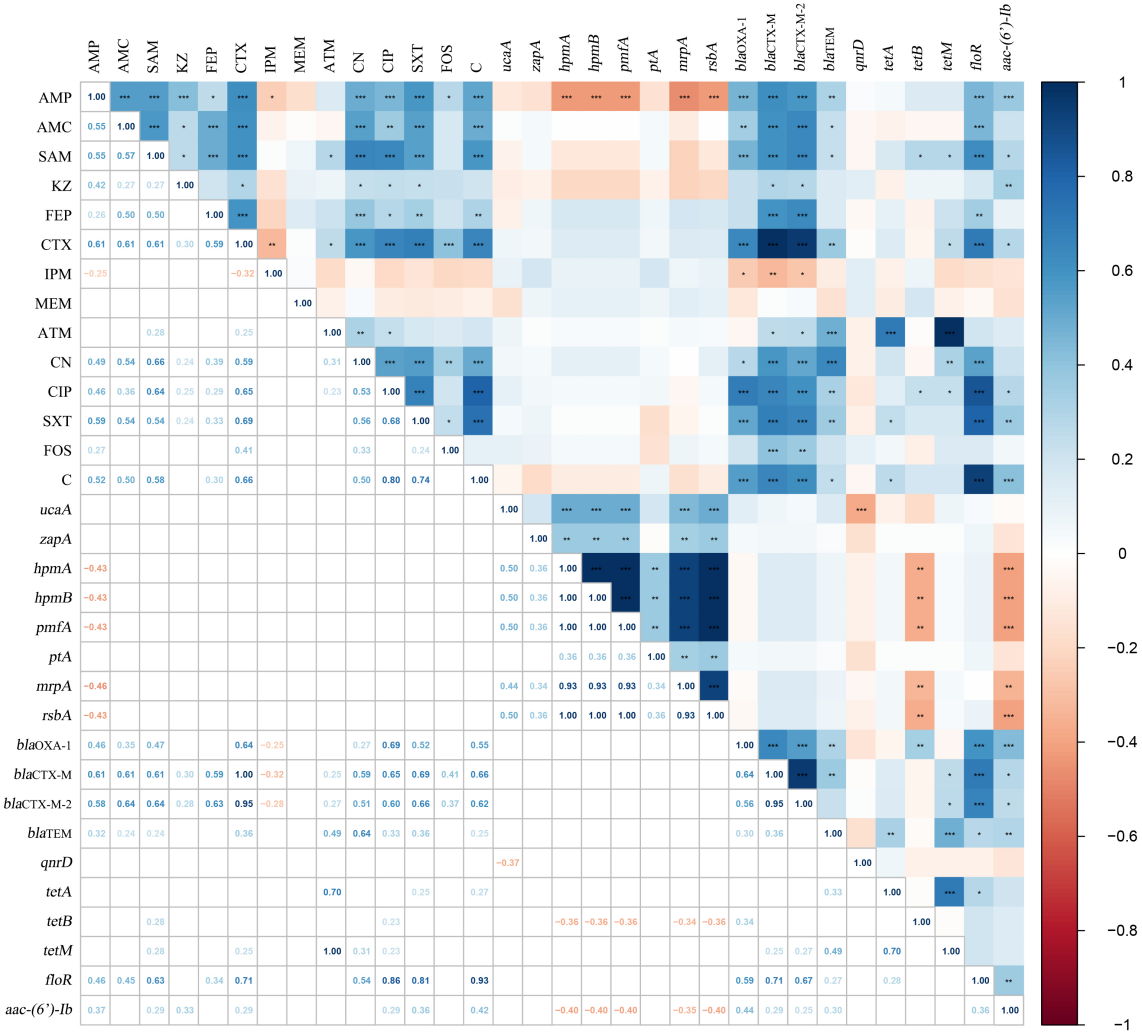
The correlations among AMR, VAGs and ARGs of *Proteus mirabilis* isolates. The numbers in the heat map represent the correlation coefficient (*r*). Positive numbers (blue) show a positive correlation and negative numbers (red) show a negative correlation.

In this study, the correlation coefficient was utilized to evaluate the relationships between these factors. Figure 3 illustrates the intra-group and inter-group correlations of AMR, VAGs, and ARGs for 75 *P. mirabilis* isolates (excluding non-statistically significant data). There were 51 pairs of the 15 antibiotics that exhibited positive correlations, with the most significant positive correlation found between CIP and C (*r* = 0.80, *p* < 0.001). Negative correlations were shown between IPM and AMP (*r* = −0.25, *p* < 0.05), and between IPM and CTX (*r* = −0.32, *p* < 0.01). However, no significant association was observed between MEM and any of the other antibiotics. Analysis of the correlation between AMR and VAGs revealed that only AMP had a negative correlation with VAGs (*hpmA*, *hpmB*, *pmfA*, *mrpA* and *rsbA*), and the most significant correlation was found between AMP and *mrpA* (*r* = −0.46, *p* < 0.001). Meanwhile, there were 67 pairs of antimicrobial and ARGs positively correlated, and 3 pairs were negatively correlated. There was the most significant correlation between CTX of β-lactam and *bla*_CTX-M_, and between ATM and *tetM* (*r* = 1.00, *p* < 0.001).

**Figure 4:**
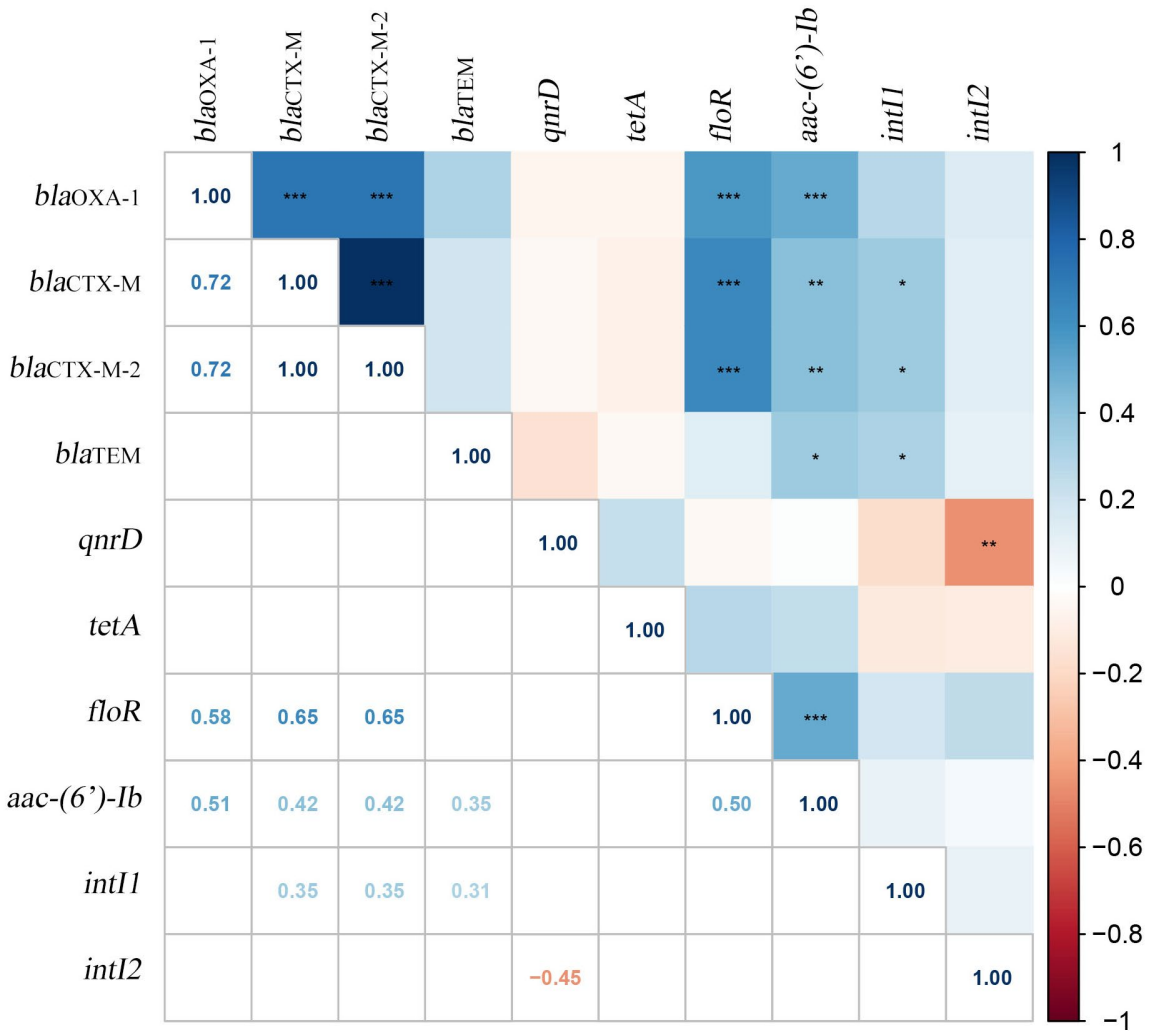
Correlation between ARGs and integrons in 40 MDR *Proteus mirabilis* strains. The numbers in the heat map represent the correlation coefficient (*r*). Positive numbers (blue) show a positive correlation and negative numbers (red) show a negative correlation.

Furthermore, 25 pairs of the 8 VAGs showed positive correlation with *hmpA*, *hpmB*, *pmfA* and *rsbA* exhibiting the strongest correlations (*r* = 1.00, *p* < 0.001). Similarly, 21 pairs of the 10 ARGs showed positive correlations, with the strongest correlation observed between *bla*_CTX-M_ and *bla*_CTX-M-2_ (*r* = 0.95, p < 0.001). Additionally, the correlation analysis between VAGs and ARGs revealed that 11 pairs of VAGs and ARGs exhibited negative correlations. Among them, *aac-(6′)-Ib* had the strongest negative correlations with *hpmA*, *hpmB*, *pmfA*, and *rsbA* (*r* = −0.40, *p* < 0.01). Specific results of the correlation analysis can be found in Figure 3. In the present study, we examined the correlation between ARGs and the integrons of 40 MDR strains (Figure 4). Our results revealed a significant positive correlation between class 1 integrons and the β-lactam-resistant genes *bla*_TEM_ (*r* = 0.31, *p* < 0.05), *bla*_CTX-M_ (*r* = 0.35, *p* < 0.05), and *bla*_CTX-M-2_ (*r* = 0.35, *p* < 0.05). It is noteworthy that a significant negative correlation was observed between *qnrD* and the class 2 integrons.

## 4. Discussion

*Proteus mirabilis* has been extensively researched as a urinary tract pathogen in humans over the past decades (Durgadevi et al., 2020; Kot et al., 2021; Wilks et al., 2021). However, there is limited research focusing on *P. mirabilis* isolated from dog feces. This study aims to investigate the prevalence and biological characteristics of *P. mirabilis* in the feces of domestic and stray dogs in Chengdu, southwestern China, to determine the potential threat it poses to public health.

Out of 241 samples of dog feces, a total of 75 non-duplicate *P. mirabilis* strains were isolated, with a prevalence of 31.12%. This prevalence is similar to that found in a study of dogs with diarrhea in Northeast China (Sun et al., 2020), where 76 out of 232 samples had *P. mirabilis* infection, accounting for 32.76%. In the same study, Sun et al. also investigated the presence of *P. mirabilis* in the feces of other animals (mink, cattle, and fowl) with diarrhea. Notably, dogs had the highest infection rate among the animals tested. In our findings, the prevalence of *P. mirabilis* in the feces of stray dogs was significantly higher than that of domestic dogs (*p* < 0.01). This may be attributed to the fact that *P. mirabilis* is commonly present in soil and sewage, and stray dogs are more susceptible to exposure to these contaminated sources, resulting in the infection of *P. mirabilis.* This agrees with previous studies conducted on non-human species, such as Namibian cheetahs and chimpanzees, suggesting that gut microbiota can be influenced by the living environment (Vilson et al., 2018; Reese et al., 2021).

The 75 *P. mirabilis* isolates from this study exhibited the abilities of swarming motility and biofilm formation. There was no significant difference in swarming motility and biofilm formation between MDR and non-MDR isolates. This finding contrasts with a study conducted by Filipiak et al., which reported that multi-drug sensitive (MDS) strains had weaker biofilm formation but stronger swarming motility compared to MDR strains (Filipiak et al., 2020). Currently, AMR and MDR of bacteria derived from animals are the focus of attention. In this study, a high resistant rate was observed for most *P. mirabilis* isolates to KZ (70.67%) and IPM (70.67%). This resistance rate was higher than that reported in other studies, such as *P. mirabilis* isolated from dogs in Japan (Harada et al., 2014) and *P. mirabilis* isolated from dogs with bacterial urine (Decôme et al., 2020). Although current research on the AMR of *P. mirabilis* isolated from dogs reports varying results, a consistent trend across these studies is the gradual increase in AMR of *P. mirabilis* over time (Decôme et al., 2020). Furthermore, differences in AMR were observed among *P. mirabilis* strains from domestic and stray dogs. Except for natural resistance to tetracycline, the highest resistance to KZ (87.80%) was found in the *P. mirabilis* isolated from domestic dogs, followed by AMP (58.54%), IPM (58.54%), SXT (51.22%) and C (51.22%). There was no significant difference in AMR to FEP, MEM, ATM, F, and FOS between *P. mirabilis* isolated from domestic and stray dogs (*p* > 0.05). For the resistance to IPM, the AMR of *P. mirabilis* isolated from stray dogs was higher than that of domestic dogs. For the rest of the antibiotics, the opposite is true. The emergence of MDR bacteria is reflected as a risk factor for public health. A total of 30 (75.00%, 30/40) MDR strains were isolated from domestic dogs, which was significantly higher than 10 (25.00%, 10/40) MDR strains isolated from stray dogs. This phenomenon may be related the widespread use (and frequent misuse) of antibiotics in clinical, and stray dogs rarely receive antibiotic treatment from veterinarians (Liu C. et al., 2021; Liu Y. et al., 2021).

Aside from antimicrobial resistance, virulence factors are also receiving considerable attention. The current investigation identified a total of 11 VAGs, with detection rates exceeding 90% for all except *mrpA* (89.33%) and *ucaA* (70.67%). Notably, the *ureC* gene was detected in 100% of isolates, which was consistent with the result that all isolates were capable of producing urease. The detection rates of VAGs in this study were found to be higher compared to a prior study conducted in Northeast China, which fecal swabs collected from diarrhea animals (dogs, mink, cattle and poultry) demonstrated *ureC* as the most prevalent VAG, observed in 90.91% of samples (Sun et al., 2020). Similar results were obtained in a study conducted on *P. mirabilis* isolated from ducks, where 100% detection rates of *ureC*, 94.3% for *rsbA*, and 91.4% for *zapA* were recorded, consistent with our findings (Algammal et al., 2021). Interestingly, a high detection rate of *ucaA* (76.47%) was observed in 34 *P. mirabilis* isolates from stray dogs was, with all other VAGs demonstrating 100% detection rates. Comparatively, *P. mirabilis* strains isolated from stray dogs exhibited a greater number of VAGs when compared to those isolated from domestic dogs. Previous research has shown that the microbiota of captive animals harbors more VAGs in comparison to that of wild animals (Guo et al., 2019; Liu C. et al., 2021). Due to increased interactions between captive animals and humans, horizontal gene transfer from other bacteria in the environment (such as air and water) leads to virulence factor accumulation (Liu C. et al., 2021). Indeed, the environmental factors surrounding stray dogs, such as exposure to rotten food, sewage and other pollutants, along with inadequate living conditions, are likely to be contributing factors to the higher incidence of VAGs detected in *P. mirabilis* isolated from these animals. In contrast, wild animals and domestic dogs may have a broader range of dietary options and cleaner lifestyles, which might result in the reduced chance of acquisition of VAGs.

In addition, the present study also involved the examination of ARGs carried by *P. mirabilis* isolates. Out of the 18 screened ARGs, 10 were detected in the 75 isolates, with *aac-(6′)-Ib* (38.67%), *qnrD* (32.00%), *floR* (25.33%), *bla*_CTX-M_ (17.33%), and *bla*_CTX-M-2_ (16.00%) being the top five ARGs detected. A Japanese study demonstated that clinical isolates of *P. mirabilis* did not exhibit positivity for *qnrA*, *qnrB*, *qnrS*, and *aac (6′)-Ib-cr*; however, 1.9% (2/105) of isolates were found to be positive for *qnrD* (Harada et al., 2014). Our study revealed higher rates of ARGs detection compared to a study from Brazil which detected only 4 ARGs (*bla*_TEM_, *bla*_SHV_, *bla*_CTX-M-1_, and *bla*_OXA-1_) in *P. mirabilis* isolated from dogs (Sfaciotte et al., 2021). However, the detection rates of ARGs in our study were lower than a previous study that detected 7 out of 14 ARGs in clinical isolates of *P. mirabilis* obtained from humanin northern Taiwan (Lin et al., 2019). The integrons system possesses the capability to capture and express exogenous ARGs. Bacteria have the perceptive capacity to acquire and aggregate ARGs from their surrounding environment through the integrase mediated by *intI* within the system, allowing for the formation of extensive drug-resistant gene arrays. Consequently, integrons assume a crucial role in the evolution of MDR bacteria and horizontal diffusion of ARGs determinants. Class 1 integrons and Class 2 integrons were detected in the MDR strain in this study, which was higher than the detection rate in a previous study on cooked meat products in China, but lower than the detection rate in Chinese human isolates (Yu et al., 2017; Lu et al., 2022). Significantly, the *P. mirabilis* isolates from domestic dogs were found to carry a significantly more ARGs compared to those isolated from stray dogs (*p* < 0.05). This observation is consistent with the result that *P. mirabilis* isolated from domestic dogs exhibited a more severe antimicrobial resistance phenotype than that isolated from stray dogs. In 2019, a study suggested that UTIs in companion animals (dogs and cats) and humans may could be caused by closely related strains of *P. mirabilis*, sharing similar ARGs in both sources (Marques et al., 2019), indicating a potential public health risk. To mitigate this risk, veterinarians must use antibiotics judiciously and avoid overuse. Additionally, researchers can explore alternative therapies such as phages and lactobacillus to combat *P. mirabilis* infections (Melo Luís et al., 2016; Shaaban et al., 2020).

A previous study showed that the occurrence and the positive correlations of VAGs and ARGs can be used as a reference for the regulatory use of antibiotics to stop the direct or indirect transmission of these resistance and virulent microbes to the natural environment (Zhang S. et al., 2021). So, we further analyzed the correlation among AMR, VAGs, and ARGs of *P. mirabilis* isolates. In this study, there was a positive correlation between the resistance phenotypes of *P. mirabilis* isolates to various antibiotics. There was also a significant positive correlation between the ARGs they carried. In addition, there was a significant positive correlation between AMR and ARGs, which were consistent with the statement of Algammal et al. (2021). It is worth noting that there were only negative correlations between AMR and VAGs, which were also reflected in the correlations between ARGs and VAGs. A previous study showed that MDR strains of *P. mirabilis* isolated from ducks did not differ significantly from the VAGs carried by non-MDR strains (Algammal et al., 2021). Therefore, our results are different from previous studies. Moreover, class 1 integrons are positively correlated with β-lactam resistance genes (*bla*_CTX-M_, *bla*_CTX-M-2_, bla_TEM_), which means that these genes may be on class 1 integrons.

## 5. Conclusion

This study has shown that *P. mirabilis* strains isolated from domestic dogs carrying fewer VAGs but more ARGs compares to those isolated from stay dogs. In addition, we observed a negative correlation between VAGs and ARGs, which is different from previous studies and requires further study at a later stage. Increased antimicrobial resistance has been detected in MDR *P. mirabilis* isolates over the past 10 years, and therefore the need to control the use of antimicrobial agents in animals to minimize the emergence and eventual spread of resistant pathogens is necessary to protect human and animal health.

## Data availability statement

The original contributions presented in the study are included in the article/Supplementary material, further inquiries can be directed to the corresponding authors.

## Author contributions

GP, LL, MD, SA, and XQ: conceptualization, methodology, and software. LL, ZD, SC, SA, and QL: data curation and writing-original draft preparation. ZZ, HL, XM, ZR, and XQ: visualization and investigation. YH, ZR, HF, and GS: supervision. LL, MD, SA, and ZZ: software and validation. LL, ZD, SC, MD, XQ, and GP: writing-reviewing and editing. All authors contributed to the article and approved the submitted version.

## Funding

This work was supported by “Research on Prevention and Control Technology and Regulation Products of Important Nutritional Metabolic Diseases of Pets,” a key Special project of “13th Five-Year Plan,” Ministry of Science and Technology (2016YFD0501009).

## Conflict of interest

The authors declare that the research was conducted in the absence of any commercial or financial relationships that could be construed as a potential conflict of interest.

## Publisher’s note

All claims expressed in this article are solely those of the authors and do not necessarily represent those of their affiliated organizations, or those of the publisher, the editors and the reviewers. Any product that may be evaluated in this article, or claim that may be made by its manufacturer, is not guaranteed or endorsed by the publisher.
