# Supplementary material for: Virulence-related factors and antimicrobial resistance in Proteus mirabilis isolated from domestic and stray dogs
Source: Front Microbiol. 2023 May 10;14:1141418. doi: 10.3389/fmicb.2023.1141418 (PMC10206225; doi:10.3389/fmicb.2023.1141418)
Supplement: Supplementary file 1 [file Data_Sheet_1.docx]

Supplementary Material

Virulence-related Factors and Antimicrobial Resistance in *Proteus mirabilis* Isolated from Domestic and Stray Dogs

Lijuan Liu ^1^, Zhiyou Dong ^1^, Shanyu Chen ^1^, Shengquan Ai ^2^, Mengyao Dong ^3^, Qianlan Li ^1^, Ziyao Zhou ^1^, Haifeng Liu ^1^, Zhijun Zhong ^1^, Xiaoping Ma ^1^, Yanchun Hu ^1^, Zhihua Ren ^1^, Hualin Fu ^1^, Gang Shu ^1^, Xianmeng Qiu ^2^*, Guangneng Peng ^1^*

*** Correspondence:** Guangneng Peng: [pgn.sicau@163.com](mailto:pgn.sicau@163.com);

Xianmeng Qiu: 13008142361@126.com

## Supplementary Figures

**
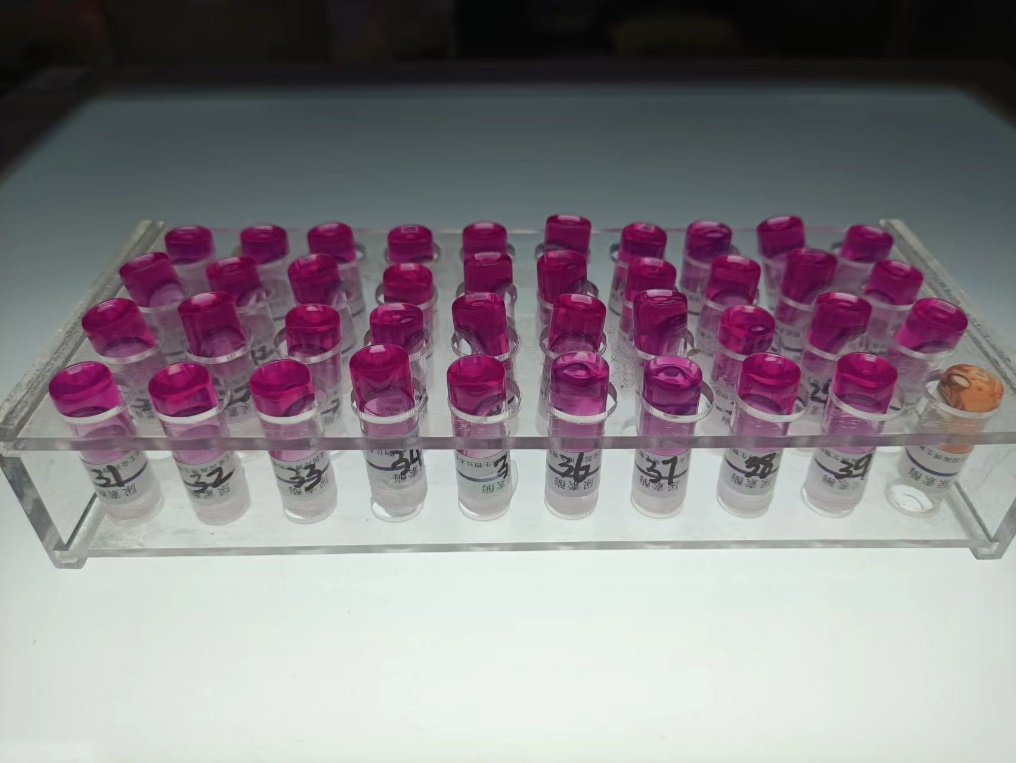
**

**Supplementary Figure 1.** **Urease production test results of *P. mirabilis* isolates.** All *P. mirabilis* isolates can produce urease.

**
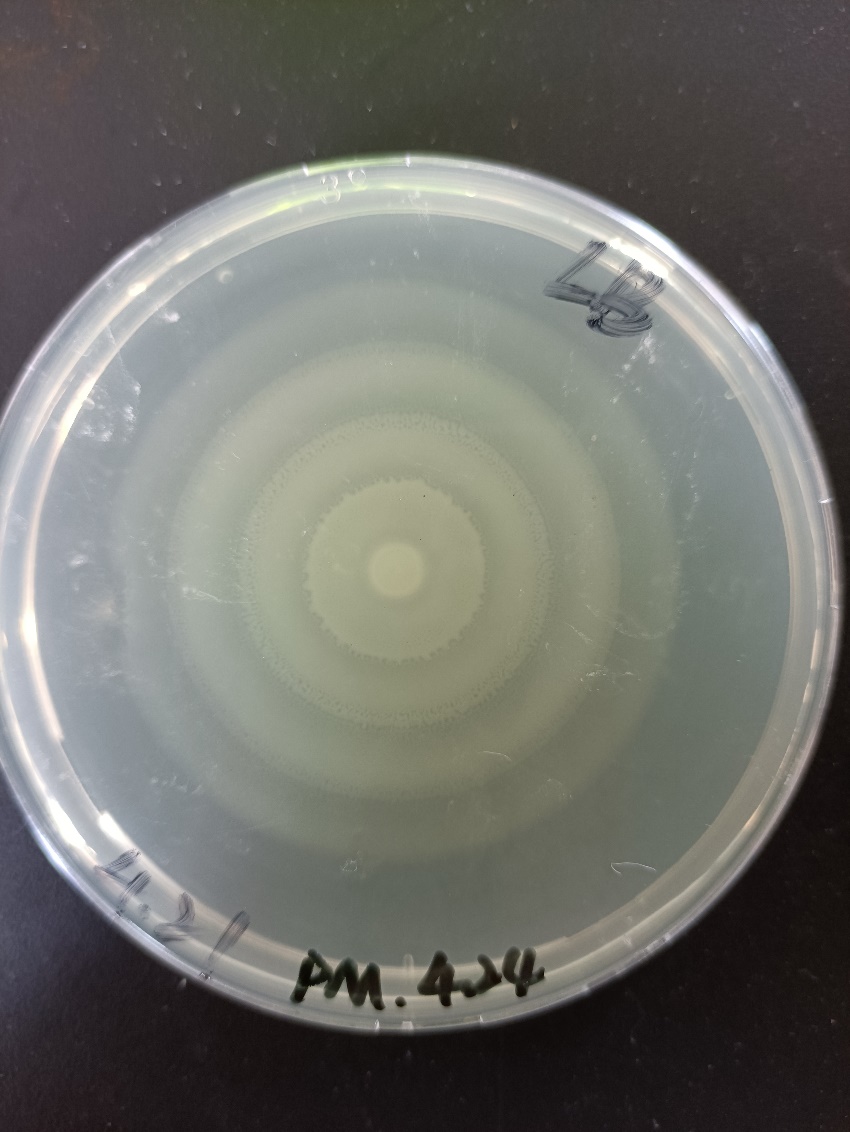
**

**Supplementary Figure 2. Swarming motility of *P. mirabilis* isolates.**


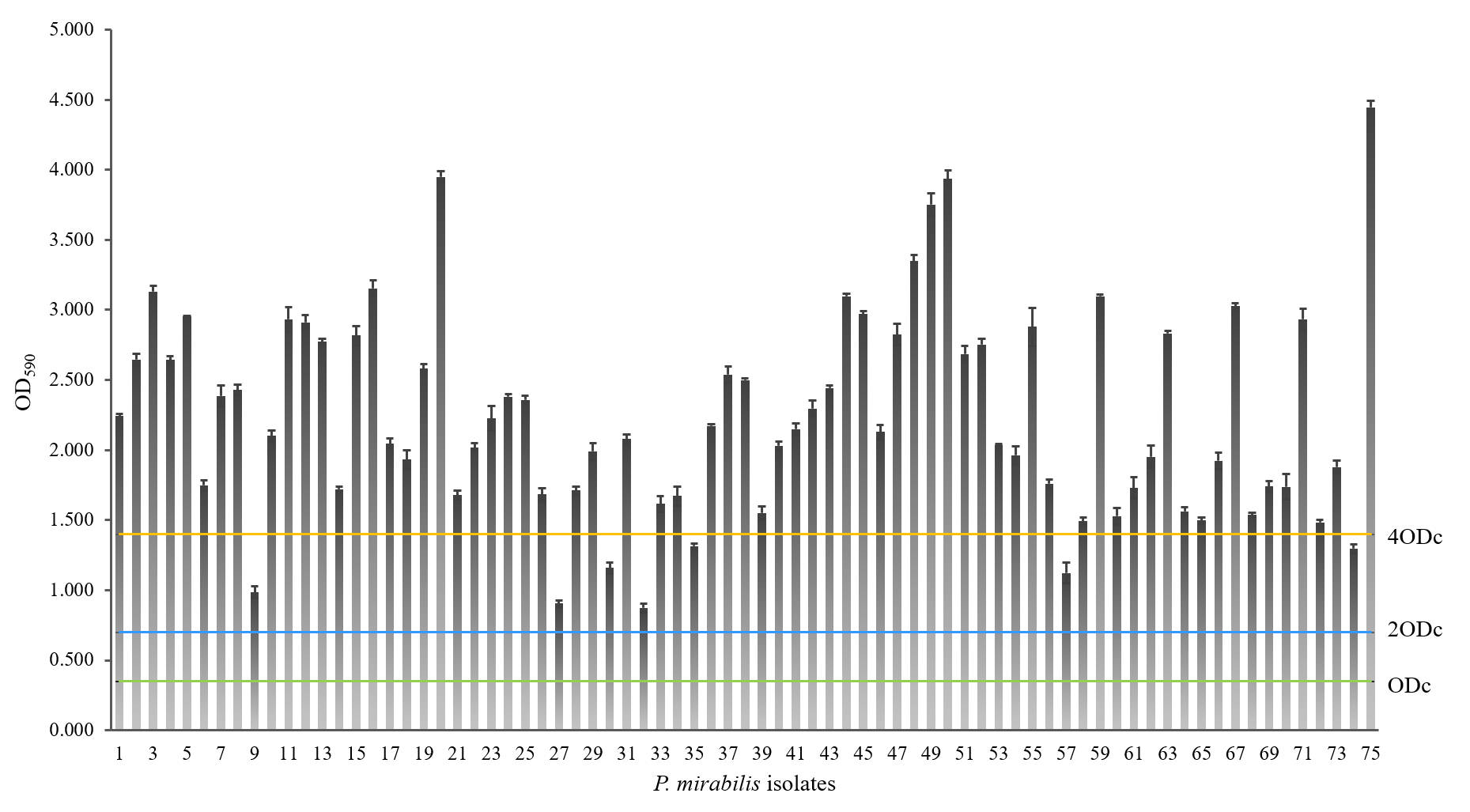


**Supplementary Figure 3.** Optical density (OD) of *P. mirabilis* isolates at 590 nm after incubation for 24h. *P. mirabilis* strains numbered 1-41 were isolated from domestic dogs, and *P. mirabilis* strains numbered 42-75 were isolated from stray dogs. The absorbance of the biofilm at 590nm was measured after crystal violet staining. The mean OD of the negative control plus three times its standard deviation (SD) was defined as the cut-off value (ODc). Based on the ODc, the biofilm-forming ability of isolates can be divided into the following four types: OD ≤ ODc is a non-biofilm-forming strain (-), ODc < OD ≤ 2ODc is a weak biofilm-forming strain (+), and 2ODc < OD≤ 4ODc is medium biofilm-forming strains (++), OD> 4ODc is strong biofilm-forming strains (+++).


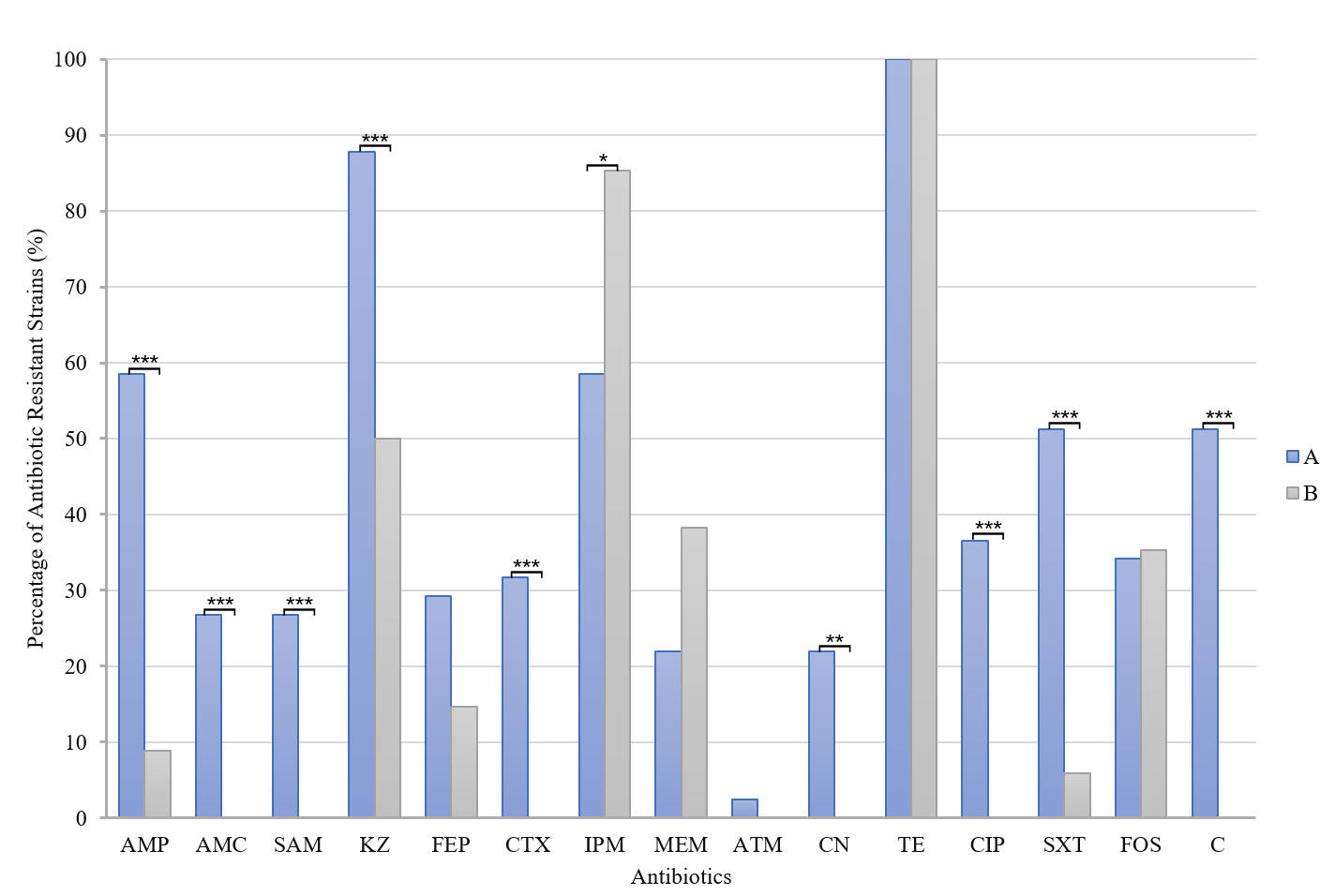


**Supplementary Figure 4. *P. mirabilis* isolated from domestic dogs and stray dogs showed different antimicrobial resistance (AMR).** A, *P. mirabilis* isolated from feces of domestic dogs; B, *P. mirabilis* isolated from feces of stray dogs; brackets, unpaired two-tailed t-test was used for a statistically significant difference; *, *P*<0.05; **, *P*<0.01; ***, *P*<0.001.

## Supplementary Tables

## Supplementary Table 1. Primer sequences, annealing temperature and expected lengths for virulence-associated genes.

| Genes | Primer Sequences (5’-3’) | Temperature (℃) | expected lengths (bp) |
| --- | --- | --- | --- |
| *fⅠiL* | F: CTCTGCTCGTGGTGGTGTCG  R: GCGTCGTCACCTGATGTGTC | 57 | 770 |
| *pmfA* | F: TCAGCATGTGGATTAGCAGCA  R: CTTGGAATTCACCTGGCGTT | 59 | 385 |
| *mrpA* | F: ATTTCAGGAAACAAAAGATG  R: TTCTTACTGATAAGACATTG | 59 | 410 |
| *ucaA* | F: GCTGGCTCATCTATGGCGTA  R: AGCGGTAGATTGTCCGGTTG | 60 | 453 |
| *ureC* | F: GTTATTCGTGATGGTATGGG  R: ATAAAGGTGGTTACGCCAGA | 58 | 317 |
| *hpmA* | F: GTTGAGGGGCGTTATCAAGAGTC  R: GATAACTGTTTTGCCCTTTTGTGC | 55 | 709 |
| *hpmB* | F: CAGTGGATTAAGCGCAAATG  R: CCTTCAATACGTTCAACAAACC | 55 | 422 |
| *zapA* | F: TGGCGCAAATACGACTACCA  R: TATCGTCTCCTTCGCCTCCA | 57 | 332 |
| *ireA* | F: AAAGGGCGAGCGATTATGTATGG  R: ATTGGCGCTATGTTTTGGTGTCA | 55 | 387 |
| *ptA* | F: CCACTGCGATTATCCGCTCT  R: ATCGGCAGAAGTGACAAGCA | 60 | 686 |

**Supplementary Table 2. Primer sequences, annealing temperature and expected lengths for antibiotic resistance genes and integrons.**

| Genes | Primer Sequences (5’-3’) | Temperature  (℃) | expected lengths (bp) |
| --- | --- | --- | --- |
| *bla*_SHV_ | F: AGCCGCTTGAGCAAATTAAAC  R: ATCCCGCAGATAAATCACCAC | 55 | 635 |
| *bla*_CMY_ | F: TGGCCGTTGCCGTTATCTAC | 55 | 868 |
| *bla*_CTX-M-2_ | R: CGTTAACGGCACGATGAC  F: ATGATGACTCAGAGCATTCG  R: CGATATCGTTGGTGGTRCCAT | 55 | 404 |
| *bla*_CTX-M_ | F: TCAAGCCTGCCGATCTGGT  R: TGATTCTCGCCGCTGAAG | 55 | 561 |
| *bla*_OXA-1_ | F: GACTTTATAAATTTAGTGTGTTTA  R: ACGTTATGAAAAACACAATACAT | 55 | 816 |
| *bla*_TEM_ | F: GGGGATGAGTATTCAACATTTCC  R: GGGCAGTTACCAATGCTTAATCA | 55 | 861 |
| *qnrA* | F: CCAGGATTTGAGTGACAGC  R: TCCCAAGGGTTCCAGCA | 65 | 592 |
| *qnrB* | F: GATCGTGAAAGCCAGAAAGG  R: ATGAGCAACGATGCCTGGTA | 65 | 476 |
| *qnrD* | F: CGAGATCAATTTACGGGGAATA  R: AACAAGCTGAAGCGCCTG | 65 | 572 |
| *qnrS* | F: GCAAGTTCATTGAACAGGGT  R: TCTAAACCGTCGAGTTCGGCG | 65 | 428 |
| *sul1* | F: GTGACGGTGTTCCGCA TTCT  R: GGTAACATTTTCGGTTCCTG | 72 | 779 |
| *sul2* | F: CATCATTTTCGGCATCGTC  R: TCTTGCGGTTTCTTTCAGC | 72 | 793 |
| *sul3* | F: CATTCTAGAAAACAGTCGTAGTTCG  R: CATCTGCAGCTAACCTAGGGCTTTGGA | 72 | 990 |
| *tetA* | F: GCTACATCCTGCTTGCCTTC  R: CATAGATCGCCGTGAAGAGG | 63 | 210 |
| *tetB* | F: TTGGTTAGGGGCAAGTTTTG  R: GTAATGGGCCAATAACACCG | 63 | 695 |
| *tetM* | F: GTGGACAAAGGTACAACGAG  R: CGGTAAAGTTCGTCACACAC | 63 | 406 |
| *floR* | F: GGCTTTCGTCATTGCGTCTC  R: ATCGGTAGGATGAAGGTGAGGA | 60 | 650 |
| *aac-(6’)-Ib* | F: ATGACCTTGCGATGCTCTATGA  R: CGAATGCCTGGCGTGTTT | 54 | 486 |
| *intI*1 | F: ACGAGCGCAAGGTTTCGGT  R: GAAAGGTCTGGTCATACATG | 56 | 525 |
| *intI*2 | F: CACGGATATGCGACAAAAAGGT  R: GTAGCAAACGACTGACGACAAAAAGGT | 56 | 789 |
| *intI*3 | F: GCCTCCGGCAGCGACTTTCAG  R: GATGCTGCCCAGGGCGCTCG | 65 | 433 |

## Supplementary Table 3. Specific antimicrobial resistance phenotypes of *Proteus mirabilis* isolates.

| Number | Antimicrobial resistance phenotypes | Number | Antimicrobial resistance phenotypes |
| --- | --- | --- | --- |
| PM1* | AMP/AMC/KZ/IPM/CN/TE/SXT/FOS | PM39 | IPM/TE |
| PM2* | AMP/KZ/IPM/TE | PM40 | KZ/IPM/TE |
| PM3* | AMP/KZ/IPM/TE | PM41 | KZ/FEP/MEM/TE |
| PM4* | AMP/AMC/SAM/KZ/FEP/CTX/IPM/MEM/CN/TE/SXT/FOS/C | PM42 | AMP/TE/ |
| PM5 | KZ/TE | PM43* | AMP/KZ/IPM/TE/FOS |
| PM6 | TE | PM44 | IPM/TE/SXT |
| PM7* | AMP/AMC/KZ/FEP/CTX/MEM/TE/SXT/C | PM45* | AMP/KZ/IPM/TE/SXT |
| PM8* | AMP/AMC/SAM/KZ/FEP/CTX/TE/CIP/SXT/FOS/C | PM46 | KZ/FEP/IPM/TE/ |
| PM9* | AMP/AMC/SAM/KZ/FEP/CTX/IPM/CN/TE/CIP/SXT/C | PM47 | IPM/TE |
| PM10* | IPM/TE/SXT/C | PM48 | IPM/MEM/TE |
| PM11 | KZ/FEP/IPM/MEM/TE | PM49 | IPM/TE/FOS |
| PM12 | KZ/TE | PM50 | IPM/TE/FOS |
| PM13 | KZ/IPM/TE | PM51 | IPM/MEM/TE |
| PM14 | KZ/IPM/TE | PM52 | IPM/TE |
| PM15* | AMP/AMC/SAM/KZ/FEP/CTX/IPM/TE/CIP/SXT/C | PM53 | KZ/IPM/TE |
| PM16 | KZ/IPM/TE/ | PM54 | KZ/MEM/TE |
| PM17* | KZ/IPM/TE/CIP/SXT/C | PM55 | IPM/TE |
| PM18* | KZ/IPM/TE/CIP/SXT/C | PM56 | IPM/TE/FOS |
| PM19* | AMP/AMC/KZ/FEP/CTX/TE/SXT/FOS | PM57 | IPM/MEM/TE |
| PM20* | AMP/AMC/SAM/KZ/IPM/CN/TE/CIP/SXT/C | PM58* | KZ/IPM/TE/FOS |
| PM21* | AMP/AMC/SAM/KZ/FEP/CTX/CN/TE/CIP/SXT/FOS/C | PM59 | IPM/MEM/TE |
| PM22* | AMP/KZ/CTX/TE/CIP/SXT/F/FOS/C | PM60 | KZ/IPM/MEM/TE |
| PM23* | AMP/SAM/KZ/FEP/CTX/IPM/MEM/CN/TE/CIP/SXT/FOS/C | PM61 | IPM/TE |
| PM24* | AMP/KZ/TE/SXT/FOS | PM62 | IPM/TE |
| PM25* | AMP/AMC/SAM/KZ/FEP/CTX/IPM/MEM/CN/TE/CIP/SXT/F/FOS/C | PM63* | KZ/IPM/TE/FOS |
| PM26* | AMP/SAM/KZ/FEP/CTX/ATM/CN/TE/CIP/SXT/FOS/C | PM64 | FEP/TE |
| PM27* | AMP/SAM/KZ/IPM/TE/F | PM65 | KZ/IPM/MEM/TE |
| PM28* | AMP/KZ/IPM/TE | PM66 | IPM/MEM/TE |
| PM29* | AMP/KZ/TE/F/FOS | PM67 | KZ/FEP/MEM/TE |
| PM30* | AMP/SAM/KZ/IPM/MEM/TE/CIP/SXT/C | PM68 | KZ/IPM/MEM/TE |
| PM31* | AMP/KZ/TE/C | PM69* | KZ/FEP/IPM/TE/FOS |
| PM32* | AMP/AMC/KZ/IPM/TE/F/C | PM70* | FEP/IPM/TE/FOS |
| PM33 | TE/CIP/F/C | PM71* | KZ/MEM/TE/FOS |
| PM34* | AMP/KZ/CTX/CN/TE/CIP/SXT/FOS/C | PM72* | KZ/IPM/MEM/TE/FOS |
| PM35* | AMP/KZ/CTX/TE/CIP/SXT/FOS/C | PM73* | KZ/IPM/TE/FOS |
| PM36* | IPM/TE/SXT/F/C | PM74 | KZ/IPM/MEM/TE |
| PM37* | KZ/IPM/MEM/TE/F | PM75* | KZ/IPM/TE/FOS |
| PM38* | KZ/MEM/TE/FOS |  |  |

*Proteus mirabilis* strains numbered 1-41 were isolated from domestic dogs, and *Proteus mirabilis* strains numbered 42-75 were isolated from stray dogs. *, "MDR" strain justified according to the definition used by Magiorakos et al (Magiorakos et al., 2012).

**Supplementary Table 4.** **The prevalence of virulence-associated genes in *P. mirabilis* isolates.**

| Virulence-associated genes | Detection rates (n) | Detection rates | | *P*-values^a^ | Detection rates | | *P*-values^b^ |
| --- | --- | --- | --- | --- | --- | --- | --- |
|  |  | Domestic dogs (n) | Stray dogs (n) |  | MDR (n) | non-MDR (n) |  |
| *ureC* | 100.00% (75/75) | 100.00% (41/41) | 100.00% (34/34) | - | 100.00% (40/40) | 100.00% (35/35) | - |
| *ucaA* | 70.67% (53/75) | 65.85% (27/41) | 76.47% (26/34) | 0.315 | 67.50% (27/40) | 74.29% (26/35) | 0.349 |
| *zapA* | 98.67% (74/75) | 97.56% (40/41) | 100.00% (34/34) | 0.547 | 97.50% (39/40) | 100.00% (35/35) | 0.533 |
| *hpmA* | 90.67% (68/75) | 82.93% (34/41) | 100.00% (34/34) | 0.011^*^ | 82.50% (33/40) | 100.00% (35/35) | 0.009^**^ |
| *hpmB* | 90.67% (68/75) | 82.93% (34/41) | 100.00% (34/34) | 0.011^*^ | 82.50% (33/40) | 100.00% (35/35) | 0.009^**^ |
| *pmfA* | 90.67% (68/75) | 82.93% (34/41) | 100.00% (34/34) | 0.011^*^ | 82.50% (33/40) | 100.00% (35/35) | 0.009^**^ |
| *FliL* | 100.00% (75/75) | 100.00% (41/41) | 100.00% (34/34) | - | 100.00% (40/40) | 100.00% (35/35) | - |
| *ptA* | 98.67% (74/75) | 97.56% (40/41) | 100.00% (34/34) | 0.547 | 97.50% (39/40) | 100.00% (35/35) | 0.533 |
| *ireA* | 100.00% (75/75) | 100.00% (41/41) | 100.00% (34/34) | - | 100.00% (40/40) | 100.00% (35/35) | - |
| *mrpA* | 89.33% (67/75) | 80.49% (33/41) | 100.00% (34/34) | 0.006^**^ | 80.00% (32/40) | 100.00% (35/35) | 0.005^**^ |
| *rsbA* | 90.67% (68/75) | 82.93% (34/41) | 100.00% (34/34) | 0.011^*^ | 82.50% (33/40) | 100.00% (35/35) | 0.009^**^ |

a, *P. mirabilis* isolated from domestic dogs were compared with *P. mirabilis* isolated from stray dogs by Fisher’s Exact test; b, MDR strains were compared with non-MDR strains by Fisher’s Exact test.

**Supplementary Table 5.** **The prevalence of antibiotic resistance genes of the *P. mirabilis* isolates.**

| Antibiotic resistance genes | Detection rates (N) | Detection rates (N) | | *P*-values^a^ | Detection rates (N) | | *P*-values^b^ |
| --- | --- | --- | --- | --- | --- | --- | --- |
|  |  | Domestic dogs (n) | Stray dogs (n) |  | MDR (n) | non-MDR (n) |  |
| *bla*_OXA-1_ | 10.67% (8/75) | 19.51% (8/41) | 0% | 0.006^**^ | 20.00% (8/40) | 0% (0/35) | 0.005^**^ |
| *bla*_CTX-M_ | 17.33% (13/75) | 31.71% (13/41) | 0% | <0.001^***^ | 32.50% (13/40) | 0% (0/35) | <0.001^***^ |
| *bla*_CTX-M-2_ | 16.00% (12/75) | 29.27% (12/41) | 0% | <0.001^***^ | 30.00% (12/40) | 0% (0/35) | <0.001^***^ |
| *bla*_TEM_ | 5.33% (4/75) | 9.76% (4/41) | 0% | 0.083 | 10.00% (4/40) | 0% (0/35) | 0.075 |
| *qnrD* | 32.00% (24/75) | 29.27% (12/41) | 35.29% (12/34) | 0.578 | 35.00% (14/40) | 28.57% (10/35) | 0.365 |
| *tetA* | 2.67% (2/75) | 4.88% (2/41) | 0% | 0.295 | 5.00% (2/40) | 0% (0/35) | 0.281 |
| *tetB* | 1.33% (1/75) | 2.44% (1/41) | 0% | 0.547 | 2.50% (1/40) | 0% (0/35) | 0.533 |
| *tetM* | 1.33% (1/75) | 2.44% (1/41) | 0% | 0.547 | 2.50% (1/40) | 0% (0/35) | 0.533 |
| *floR* | 25.33% (19/75) | 46.34% (19/41) | 0% | <0.001^***^ | 45.00% (18/40) | 2.86% (1/35) | <0.001^***^ |
| *aac-(6’)-Ib* | 38.67% (29/75) | 65.85% (27/41) | 5.88% (2/34) | <0.001^***^ | 55.00% (22/40) | 20.00% (7/35) | 0.002^**^ |

a, *P. mirabilis* from domestic were compared with *P. mirabilis* from stray dogs by Fisher’s Exact test; b, MDR strains were compared with non-MDR strains by Fisher’s Exact test.

## Other Supplementary Material

**GenBank accession number(s) of 75 *Proteus mirabilis* isolated:**

| The number of *P. mirabilis* isolates | Accession number | The number of *P. mirabilis* isolates | Accession number |
| --- | --- | --- | --- |
| PM 1 | OP393031 | PM39 | OP393090 |
| PM2 | OP393032 | PM40 | OP393091 |
| PM3 | OP393033 | PM41 | OP393092 |
| PM4 | OP393034 | PM42 | OP393093 |
| PM5 | OP393035 | PM43 | OP393094 |
| PM6 | OP393036 | PM44 | OP393095 |
| PM7 | OP393037 | PM45 | OP393096 |
| PM8 | OP393038 | PM46 | OP393097 |
| PM9 | OP393039 | PM47 | OP393098 |
| PM10 | OP393040 | PM48 | OP393099 |
| PM11 | OP393041 | PM49 | OP393100 |
| PM12 | OP393042 | PM50 | OP393101 |
| PM13 | OP393043 | PM51 | OP393102 |
| PM14 | OP393044 | PM52 | OP393103 |
| PM15 | OP393045 | PM53 | OP393104 |
| PM16 | OP393046 | PM54 | OP393105 |
| PM17 | OP393047 | PM55 | OP393051 |
| PM18 | OP393048 | PM56 | OP393052 |
| PM19 | OP393049 | PM57 | OP393053 |
| PM20 | OP393050 | PM58 | OP393054 |
| PM21 | OP393072 | PM59 | OP393055 |
| PM22 | OP393073 | PM60 | OP393056 |
| PM23 | OP393074 | PM61 | OP393057 |
| PM24 | OP393075 | PM62 | OP393058 |
| PM25 | OP393076 | PM63 | OP393059 |
| PM26 | OP393077 | PM64 | OP393060 |
| PM27 | OP393078 | PM65 | OP393061 |
| PM28 | OP393079 | PM66 | OP393062 |
| PM29 | OP393080 | PM67 | OP393063 |
| PM30 | OP393081 | PM68 | OP393064 |
| PM31 | OP393082 | PM69 | OP393065 |
| PM32 | OP393083 | PM70 | OP393066 |
| PM33 | OP393084 | PM71 | OP393067 |
| PM34 | OP393085 | PM72 | OP393068 |
| PM35 | OP393086 | PM73 | OP393069 |
| PM36 | OP393087 | PM74 | OP393070 |
| PM37 | OP393088 | PM75 | OP393071 |
| PM38 | OP393089 |  |  |
